# Clinical outcome of non-surgical root canal treatment using different sealers and techniques of obturation in 237 patients: A retrospective study

**DOI:** 10.1007/s00784-024-05871-4

**Published:** 2024-08-10

**Authors:** Mateusz Radwanski, Krystyna Pietrzycka, Tan Fırat Eyüboğlu, Mutlu Özcan, Monika Lukomska-Szymanska

**Affiliations:** 1 Department of Endodontics, Medical University of Lodz, Lodz, Poland; 2 Department of Endodontics, Faculty of Dentistry, Istanbul Medipol University, Istanbul, Türkiye; 3 Clinic of Masticatory Disorders and Dental Biomaterials, Center for Dental Medicine, University of Zurich, Zurich, Switzerland; 4 Department of General Dentistry, Medical University of Lodz, Lodz, Poland

**Keywords:** Bioceramic sealer, Clinical outcome, Clinical trial, Dental materials, Epoxy-resin-based sealer, PAI index, Retrospective study

## Abstract

**Objectives:**

The aim of this retrospective study was to compare the clinical results of two root canal sealers and three obturation techniques used for non-surgical root canal treatment.

**Materials and methods:**

A total of two hundred eighty-three root canal treated teeth in two hundred thirty-seven patients with minimum a 6-month follow-up was included for this study. The canals were filled with three different modes: 1) cold lateral condensation (CLC) and AH Plus Sealer; 2) continuous wave condensation technique (CWC) and AH Plus Sealer, and 3) sealer-based obturation technique (SBO) and AH Plus Bioceramic Sealer. The treatment outcome was analysed based on clinical signs and symptoms, and periapical radiograph (periapical index, PAI).

**Results:**

There were no significant differences in treatment outcome between various sealers and filling techniques applied. The sealer extrusion was found most frequently in the CWC group (60.67%), followed by SBO (59.21%) and CLC (21.19%) with statistically significant differences (p < .05). The initial diagnosis, previous treatment and sealer extrusion (p < .05) were prognostic factors that affected treatment outcome.

**Conclusions:**

Based on the findings of this study, neither the sealer type nor the filling technique affected the treatment success while preoperative diagnosis, previous treatment and sealer extrusion had significant effect on the outcome.

**Clinical relevance:**

A bioceramic sealant applied along with the single-cone technique might be considered as an alternative method in root canal obturation.

## Introduction

Root canal obturation is crucial in endodontic treatment, preventing reinfection and promoting healing. Gutta-percha and sealers are used for filling canals [1]. So far, gutta-percha, as a core, and sealers have been used for filling canals. Epoxy-resin-based sealers (ERBSs) are the most popular sealers [2], which are considered the “gold standard” due to their physicochemical and antibacterial properties [3, 4]. On the other hand, recently introduced calcium-silicate-based sealers (CSBSs) provide biocompatibility, promotion of hard tissue formation, antibacterial properties, higher bond strength to dentin than ERBSs, and superior seal [5]. Previous studies presented better dentinal tubule penetration of CSBSs than ERBSs [6–8] whereas other studies concluded comparable sealing [9] and healing properties [10] between both sealer types. The results regarding the toxicity are contradictory; some studies indicate low geno- and cytotoxicity of CSBSs and ERBSs [11, 12]. On the other hand, the genotoxicity of bioceramic materials, i.e. premixed BioRoot RCS was reported, while other ready to use CSBSs (AH Plus Bioceramic Sealer or Total Fill BC Sealer) did not show any effect on genome destabilization [13]. It should be noted that the toxicity of the material may be influenced by sealer compositions, release of substances during the setting and subsequent dissolution of the material and percentages of bioactive components [13].

Numerous techniques have been put forth for introducing gutta-percha into root canals. Cold lateral condensation (CLC) is a widely used technique [14] for introducing gutta-percha into root canals, offering low cost, short learning curve, and controlled placement [15]. However, it lacks adaptation to root canal walls, fills canal irregularities, and may cause homogeneity issues [14]. Excessive forces can also lead to root defects and fractures [16].

Sealer-based obturation (SBO) is a technique where a single-cone is inserted into the canal after sealer application [17], resulting in a larger amount of sealer and voids [18]. This method is less time-consuming, simple, and accessible, but may lead to pore formation, solubility of CSBS in tissue fluids, and resorption over time [19, 20].

Continuous wave of condensation is a two-step warm vertical condensation technique, superior to SBO and CLC in hermetic obturation [21]. It requires clinical training and special equipment, and larger root canal preparation size and taper which in return may initiate cracks and vertical root fractures [22].

The success of a root canal treatment operation depends not only on the clinician’s technical proficiency and expertise but also on the materials and technique employed [23]. Selecting the appropriate filling technique and sealer can be challenging for a practitioner due to the wide range of options and variations. Although bioceramic materials are widely accessible, there is little research evaluating their therapeutic efficacy in the literature [24–30]. Scarce clinical research in this field reports no differences in healing, post-operative pain and apical extrusions between ERBSs and CSBSs [31–34].

The aim of this retrospective study was to compare clinical results of three obturation techniques used for non-surgical endodontic treatment. The null hypothesis was that there would be no statistically significant differences in treatment outcomes between different root canal sealers and filling techniques applied.

## Materials and methods

The present study was approved by local ethics committee (RNN/290/23/KE; 12.12.2023). The sample size was determined by assessing previous similar research and calculated with a significance level of 5%. The statistical power of 80% resulted in estimation of 150 teeth (50 teeth per group) (G*Power software ver. 3.1.9.7 for Windows; Heinrich-Heine-Universität Düsseldorf, Düsseldorf, Germany; http://www.gpower.hhu.de/) [26, 35].

### Inclusion and exclusion criteria

The inclusion criteria were as follows: patients over 18 years old that had at least one permanent single or multi-rooted mature tooth with signs or symptoms indicating the need for endodontic treatment (primary or secondary) and a minimum follow-up period of 6 months. Additionally, the subjects with good general medical condition (ASA I and II) were included with acceptable quality of preoperative, postoperative and follow up radiographs and records.

The exclusion criteria were relevant medical history and chronic diseases. The teeth with internal or external root resorption, or with evidence of perforation were excluded. Moreover, the presence of endodontic-periodontal lesions (severe periodontal bone loss), underfilled root canals (> 2 mm from the radiological apex), and evidence of vertical root fracture (a narrow deep probing defect and/or a J-shaped lesion with a previously treated teeth) disqualified teeth from evaluation.

After the enrolment of patients, the diagnosis process included clinical (history of pain, responses to sensitivity test, palpation and percussion) and radiographical examination (periapical X-ray showing at least the full root(s) and approximately 2–3 mm of periapical region). Before commencement of treatment, patients’ demographics, such as age and gender, were recorded.

### Root canal treatment protocol

All endodontic procedures were performed according to the guidelines of the European Society of Endodontology (ESE) [36]. After access preparation, the canals were instrumented under a dental microscope (Zeiss Extaro 300, Carl Zeiss, Gőttingen, Germany) and rubber dam isolation by one operator (M.R.). For primary treatments, root canals negotiation and working length determination were performed with the use of the C-pilots (sizes: 06–10) (VDW GmbH, Munich, Germany). For the secondary root canal treatments, previous obturation materials (GP and sealer) and canals obstruction were removed with a combination of ultrasonics, and rotary instruments HyFlex™ Remover (Coltene-Whaledent, Allstetten, Switzerland); the canals were then renegotiated by hand with the C-pilots. In all cases, the working length was determined using an electronic apex locator, Woodpex V (Guilin Woodpecker Medical Instruments Co., Ltd., China) and confirmed with a radiograph [36]. For root canal shaping, in both types of treatment, the following files were used: Path Files [sequence: #1 (13/0.02); #2(16/0.02)] (Dentsply Sirona Endodontics, Ballaigues, Switzerland) – as glide path, ProTaper Next files [sequence: X1 (17/0.04), X2 (25/0.06), X3 (30/0.07), X4 (40/0.06) and X5 (50/0.06)] (Dentsply Sirona Endodontics, Ballaigues, Switzerland). All files were applied according to the manufacturer’s instructions (300 rpm and torque 2.5 Ncm) using an X-smart Endodontic Motor (Dentsply Sirona Endodontics, Ballaigues, Switzerland). All canals were shaped with a minimum number of two files and a maximum number of five files, depending on the root canal size, which was decided by the operator.

After each file, copious amounts of irrigation with 5 mL of 5.25% NaOCl (CHLORAXiD, Cerkamed, Stalowa Wola, Poland) were applied. Next, the following rinsing protocol was implemented: 2.5 mL physiological saline for 5 min., 5 mL 40% citric acid (Cerkamed, Stalowa Wola, Poland) for 1 min., 2.5 mL physiological saline for 5 min., and 5 mL 5.25% NaOCl for 5 min., followed by 2.5 mL of physiological saline for 5 min. For irrigation, 5 mL disposable plastic syringes with 30-gauge Endo-Eze Tips (Ultradent, South Jordan, UT, USA) were introduced 1 mm shorter than their working length. The EDDY—Endo Irrigation Tip (VDW GmbH, Munich, Germany) was used for irrigation activation. The gutta-percha cones were inserted to the full working length utilizing the tug-back feeling before the canals were dried with paper points (Dentsply Sirona Endodontics, Ballaigues, Switzerland). Various treatment modalities were used to fill the canals (Table 1).

**Table 1:** The filling techniques and sealers used in the present study

Group	Obturation technique	Sealer
CLC + AH Plus	Cold lateral condensation (CLC)	AH Plus Sealer
CWC + AH Plus	Continuous wave condensation technique (CWC)	AH Plus Sealer
SBO + AH Plus Bioceramic Sealer	Sealer-based obturation technique (SBO)	AH Plus Bioceramic Sealer

In the CLC group, the sealer (AH Plus) was introduced into the canal on the master cone using two or three vertical pumping movements. Then, the appropriate spreader (spreaders 20–40, Mani, Inc., Japan) was selected, given that it should reach 1–2 mm shorter than the working length. The accessory cones in a corresponding size or one size smaller than the chosen spreader were used for condensation. The filling was continued until the canal orifice was reached, and then the gutta percha was cut off with the gutta-percha cutter C-Blade (Pol-Intech, Lodz, Poland). Next, a cold plugger was used for gutta-percha vertical compaction at the canal orifice (Machtou Pluggers; Dentsply Sirona Endodontics, Ballaigues, Switzerland).

In the CWC group, gutta-percha cones were coated with the AH Plus sealer and inserted into the root canal. A heat plugger (Fast Pack Plugger Tips, E-Connect Eighteenth, China) heated up to 200 °C/392°F was applied to cut gutta-percha. The selected Fast Pack tip reached 4–5 mm short of the working length, gave a snug fit at the tip, and was used for compaction. The rest of the canal was filled with injected, thermoplasticized gutta-percha (180 °C/356°F) using Fast Fill (E-Connect Eighteenth, China) and appropriate cold pluggers (Machtou Pluggers, Dentsply Sirona Endodontics, Ballaigues, Switzerland) matching the diameter of the canal orifice were used.

In the SBO group, AH Plus Bioceramic Sealer (Denstply DeTrey GmbH, Konstanz, Germany) was introduced with 24-gauge tip and pushed using the gutta-percha cone with up-and-down movements and gentle rotation for better sealer penetration as described by manufacturer. In the case of wide canals, 1 or a maximum 2 additional cones were added to optimize sealing. Then, the filling was cut off with the Gutta percha cutter C-Blade at the orifice level and condensed using cold pluggers (Machtou Pluggers, Dentsply Sirona Endodontics, Ballaigues, Switzerland).

The pulp chambers of all treated teeth were cleaned and temporarily filled with Teflon and glass-ionomer Fuji IX (GC Europe, Leuven, Belgium), and then the control X-rays were performed. Subsequently, the final restoration was performed on the next visit.

After the treatment, a periapical X-ray was analysed in terms of quality of root canal obturation: sealant extrusion, homogeneity, and level of root filling (adequate, long). In multi-rooted teeth, sealer extrusion was noted when it was detected at least at one root. In the present study, the extrusion of gutta-percha beyond the radiographic apex was classified as an extruded filling. The quality of root canal filling was blindly assessed by two independent observers, endodontists (K.P. and M.R.). Initial calibration, before the evaluation process, was performed on 30 randomly selected cases not included in the present study. During the study subjects evaluation, for inter-examiner agreement, the kappa score (K) was 0.80, and for intra-examiner agreement, it was 0.88 and 0.90, respectively; both indicating very good agreement [37]. In the event of disagreement, the case was discussed until a consensus was reached.

### Outcome of the treatment

The patients were recalled for control at least 6 months after treatment for clinical and radiographic examination. Pain, swelling, sinus tract, periodontal pocket, or any history of pain were recorded. The type of final restoration (direct/indirect) was noted. The group of direct restorations included composite fillings, while indirect restorations included posts, crowns and fixed bridges.

Pre-treatment and follow-up X-rays were analysed by endodontists (K.P., and M.R.). Additionally, the periapical index (PAI index) was noted according to the scale:

- PAI 1: Normal periapical bone structure.
- PAI 2: Small changes in bone structure with no demineralization.
- PAI 3: Changes in bone structure with some diffuse mineral loss.
- PAI 4: Apical periodontitis with well-defined radiolucent area.
- PAI 5: Severe apical periodontitis with exacerbating features.

The highest PAI value of all records was noted in multi-rooted teeth. Teeth were classified into three outcome categories. The clinical assessment was "success" for the healed and healing categories and "failure" for not-healed teeth based on loose criteria. According to strict criteria, only healed cases were classified as "success”. The examples of each category are presented in Table 2.

**Table 2:**
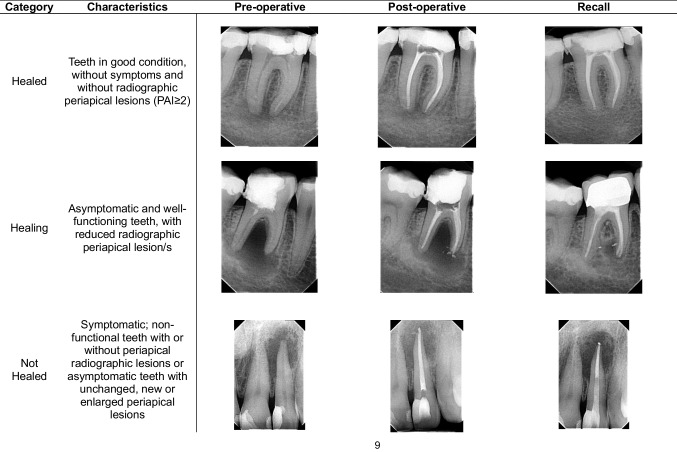
Characteristics of outcome categories. Pre-operative, post-operative and recall periapical X-rays for healed, healing and not healed cases

### Statistical analysis

The normality of the data was confirmed with the Kolmogorov–Smirnov test, and then Kruskal–Wallis’s test was performed for independent sample analysis. The influence of different variables on the outcomes of the treatment was statistically compared with χ^*2*^ test or Fisher exact test, followed by multivariate analysis with logistic regression. All statistical analyses were conducted with the statistical software package Statistica v. 13.3 (StatSoft, Inc., OK, USA), and statistical significance was set at *p* < 0.05.

## Results

### Demographic characteristics

A total of two hundred thirty-seven patients were included in the analysis, with an average age of 45.28 years (± 15 years). As a result, two hundred eighty-three teeth were treated, mostly posterior teeth (87.63%), and mostly primary endodontic treatment (61.48%). The mean follow-up time amounted to 7.56 ± 3.9 months, while the minimum follow-up was 6 months and the maximum was 30 months. The characteristics of the patient pool are summarized in Table 3.

**Table 3:** Demographic features of the patient pool

Characteristics		*n* (%)
Sex	Male	106 (44.73%)
	Female	131 (55.27%)
Average age		45.28 years
Treatment type	Initial	174 (61.48%)
	Retreatment	109 (38.52%)
Tooth type	Maxillary anterior	21
	Maxillary posterior	127
	Mandibular anterior	14
	Mandibular posterior	121
Average time to recall		7.56 months

### PAI index before treatment and the average time to recall

The average PAI index before treatment was 2.33 for CLC + AH Plus, 2.29 for CWC + AH Plus, and 2.79 for SBO + AH Plus Bioceramic Sealer, respectively. The average time to recall was 7.96 months for CLC + AH Plus; 7.08 months for CWC + AH Plus, and 7.50 months for SBO + AH Plus Bioceramic Sealer, separately. The analysis showed no statistically significant differences between the compared groups in terms of PAI and time to recall (p > 0.05).

### The treatment outcome based on the loose and strict criteria

Based on loose criteria, the sealers used in the study (AH Plus and AH Plus Bioceramic Sealer) did not significantly influence the treatment outcome (success rate: 94.2% and 94.74%, respectively). In sixteen cases (12 for AH Plus and 4 for AH Plus Bioceramic Sealer), patients reported signs and symptoms, and periapical X-ray analysis revealed treatment failure. The CLC, CWC, and SBO obturation techniques showed 94.07%, 94.38% and 94.74% success rate, respectively. Additionally, no statistical differences between filling methods were observed (p = 0.98064). Sealer extrusion was found most frequently in CWC (60.67%; 54/89), then in SBO (59.21%; 45/76) and CLC (21.19%; 25/118). Sealer extrusion appeared significantly more often in CWC and SBO when compared to CLC (p < 0.05). The periapical lesion was not statistically associated with a greater risk of filling material extrusion when compared to the absence of radiolucency (p > 0.05). None of the evaluated factors significantly influenced the treatment outcome in terms of loose criteria (p > 0.05), and multivariate analysis using logistic regression did not identify any significant predictors.

According to strict criteria, teeth with vital pulp exhibited the highest success rate (96.70%; 88/91), followed by necrotic teeth (83.13%; 69/83) and retreated teeth (82.57%; 90/109), with significant differences between groups (p = 0.00465). The primary treatment was associated with a statistically greater success rate when compared to retreated cases (p = 0.04595). The sealer extrusion significantly reduced the success rate in comparison to teeth without extrusion (p = 0.02518). The CLC showed a higher success rate (92.37%) when compared with other filling techniques (CWC: 82.02% and SBO: 85.53%); hence, it was not statistically significant (p = 0.07491). Multivariate analysis showed that, when strict criteria were considered, secondary treatment, necrotic cases, and sealer extrusion may delay healing and contribute to treatment failure. The influence of different predictors on treatment outcomes is presented in Table 4.

**Table 4:** The influence of different predictors on treatment outcomes based on loose and strict criteria. The bold font indicates the statistical significance

	Predictor	Loose criteria			Strict criteria		
	Total (*n* = 283)	Success (*n* = 267)	Failure (*n* = 16)	*P* value	Success (*n* = 247)	Failure (*n* = 36)	*P* value
Sex	Male (123)	116 (94.31%)	7 (5.69%)	.980	108 (87.80%)	15 (12.20%)	.815
	Female (160)	151 (94.38%)	9 (5.62%)		139 (86.88%)	21 (13.13%)	
Age (y)	≤ 50 (177)	168 (94.92%)	9 (5.08%)	.592	157 (88.70%)	20 (11.30%)	.353
	> 50 (106)	99 (93.40%)	7 (6.60%)		90 (84.91%)	16 (15.09%)	
Tooth type	Anterior (35)	34 (97.14%)	1 (2.86%)	.589	29 (82.86%)	6 (17.14%)	.447
	Premolar (68)	65 (95.60%)	3 (4.40%)		62 (91.18%)	6 (8.82%)	
	Molar (180)	168 (93.33%)	12 (6.67%)		156 (83.33%)	24 (16.67%)	
Vitality	Vital (91)	88 (96.70%)	3 (3.30%)	.460	88 (96.70%)	3 (3.30%)	**.004**
	Necrotic (83)	78 (93.98%)	5 (6.02%)		69 (83.13%)	14 (16.87%)	
	Retreatment (109)	101 (92.66%)	8 (7.34%)		90 (82.57%)	19 (17.43%)	
Treatment type	Primary (174)	166 (95.40%)	8 (4.60%)	.331	157 (90.23%)	17 (9.77%)	**.045**
	Secondary (109)	101 (92.66%)	8 (7.34%)		90 (82.57%)	19 (17.43%)	
Sealer extrusion	Present (124)	118 (95.16%)	6 (4.84%)	.600	102 (82.26%)	22 (17.74%)	**.025**
	Absent (159)	149 (93.71%)	10 (6.29%)		145 (91.19%)	14 (8.81%)	
Filling length	Normal (274)	259 (94.53%)	15 (5.47%)	.471	241 (87.96%)	33 (12.04%)	.059
	Long (9)	8 (88.89%)	1 (11.11%)		6 (66.67%)	3 (33.33%)	
Filling Technique	CLC (118)	111 (94.07%)	7 (5.93%)	.980	109 (92.37%)	9 (7.63%)	.074
	CWC (89)	84 (94.38%)	5 (5.62%)		73 (82.02%)	16 (17.98%)	
	SBO (76)	72 (94.74%)	4 (5.26%)		65 (85.53%)	11 (14.47%)	
Sealer	AH Plus (207)	195 (94.20%)	12 (5.80%)	.863	181 (87.44%)	26 (12.56%) 10	.893
	AH Plus Bioceramic (76)	72 (94.74%)	4 (5.26%)		66 (86.84%)	(13.16%)	
Final Restoration	Direct Restoration (209)	197 (94.26%)	12 (5.74%)	.914	180 (86.12%)	29 (13.88%)	.327
	Indirect restoration (74)	70 (94.60%)	4 (5.40%)		67 (90.54%)	7 (9.46%)	

## Discussion

The null hypothesis was accepted in light of the current study's findings since, irrespective of the filling technique and sealer used, no statistically significant variations in the treatment outcome were discovered based on neither loose nor strict criteria. Previous studies evaluating other CSBS materials reported similar outcome results compared to our results [24, 28, 38]. The comparison between CSBS and ERBS (AH Plus) also presented comparable results with significant difference on the success rate to root canal treatment [25, 27]. It is important to note that the significant degree of variability in terms of differences in methodology, such as clinical process, type of assessment, and number of studied cases, should be taken into consideration when making direct comparisons with other studies. On the other hand, successful treatment outcomes demonstrated that examined root canal sealers can produce comparable therapeutic effects irrespective of the treatment method. As was confirmed by other research [25], the obturation technique (CLC, CWC, and SBO) had no discernible impact on the treatment outcome in the current investigation.

Compared to a previous study the success rates for each group presented similar results based on the loose criteria, while superior results based on the strict criteria [25]. The discrepancy in strict criteria between studies may be due to the differences in sample size, evaluation period, and methodologies. In contrast to the present study, others reported that the warm technique was significantly superior or equal to CLC [39, 40], but traditional sealers (zinc-oxide sealers and ERBS) were applied in the latter research, therefore, direct comparison with CSBS was not possible. However, the randomized study, which compared the single cone and BioRoot RCS with zinc-oxide sealer (Pulp Canal Sealer™ EWT; Kerr Corporation, Orange, CA) with warm technique, reported the similar survival rate and progressive decrease in PAI after 12-month follow-up [41]. Additionally, healing of apical periodontitis at the first-year follow-up was slightly, but not significantly better in the CBCS group than warm vertical compaction with GP and ZOE sealer [41].

The research reveals that treatment prognostic factors include preoperative diagnosis, secondary treatment, and postoperative sealer extrusion. The success rate of endodontic treatment in vital teeth is 84%, while non-vital pulps and periapical radiolucency are 75% [42]. Preoperative diagnosis do not significantly affect treatment outcomes but may affect healing delay in necrotic and retreated cases [43]. Periapical healing is mainly related to the size of the lesion before treatment [44].

The kind of treatment (primary or secondary) had no bearing on the treatment outcome based on the loose criteria, which was in line with earlier research [24, 39]. The overall primary treatment success rate in the current data was 95.4%, and the secondary rate was 92.66%. The lack of difference between these two types of treatment may be the result of a short follow-up period. Hence, some studies with medium or long follow-up period reported significantly higher healing rate for initial endodontic treatments than for nonsurgical retreatments [45, 46].

Strict criteria revealed that retreatment's success rate was substantially lower (82.57%, p < 0.05) than that of primary treatment (90.23%). Positive secondary treatment results were found in a systematic study by Sabeti et al., with periapical healing and success rates of 78.8% and 78.0%, respectively, under rigorous criteria, and 87.5% and 86.4%, respectively, under loose criteria [47].

Unintentional sealer extrusion can result from apical constriction issues, inflammatory resorption, immature apex, or incorrect working length [29]. It can delay or prevent healing while the periapical response is greatly influenced by the composition and extrusion amount of the sealer [28, 39, 42, 44]. Over time, extruded material can resorb or remain in the tissue [27]. CSBS persists in periapical tissues due to low solubility, but its bioactivity favours hydroxyapatite formation and bone replacement [24]. It is worth mentioning that acidic pH associated with periapical lesions may significantly worsen setting of the extruded bioceramic sealers [48].

Existing literature addressing the correlation between extrusion of root canal sealers and healing outcome is not entirely consistent. A previous systematic review analysis, limited to 2-year recall, revealed that sealer extrusions could have a negative effect on root canal treatment outcome [49]. In addition, another study reported a 32% higher risk of non-healing in case of extrusions when compared with cases without presence of the sealer in the periapical tissues [50]. Therefore, some authors claim that avoiding filling material extrusion is crucial for optimal healing outcomes [42]. On the contrary, several retrospective cohort and meta-analyses have shown that the presence of extruded sealer may not affect overall results [24, 30, 51]. The reported overfilling with use of epoxy resin sealers revealed no impact on treatment outcome [51–53]. Similarly, CSBSs overfills had no significant effect on the final results [24, 28].

The extrusion of the sealer into the periapical tissues was more often observed in case of thermal methods and CSBS applied along with the single-cone technique due to the increased flowability of the materials [28, 42]. These observations were in accordance with the present results, in which significantly less sealer extrusion was observed in CLC than in CWC and SBO. Hence, it was claimed that extrusion might be associated with the presence of lesions [24]. This observation was not confirmed in the present study, where sealer-beyond-apex was not statistically correlated with the presence of apical radiolucency.

The present study did not show any statistically significant differences between root canal sealers and placement techniques. In the present study, the overall success rate amounted to 94.35% based on the loose criteria, while according to strict criteria, the overall success was 87.28%. Additionally, the systematic review found no difference between zinc oxide sealer and ERBS in non-surgical treatment outcome, additionally the therapeutic success was comparable to the present study (86.5% and 87.35%, respectively) [25]. Also, other sealers, namely glass-ionomer sealer, showed to be successful in 94.4% of cases [54]. Furthermore, another systematic review showed no differences in the success rate of primary non-surgical endodontic treatments when the cold lateral compaction technique and other obturation techniques (single-cone, carrier based obturation, warm vertical compaction) were applied [55]. Moreover, there was no difference observed between procedures (non-lateral compaction technique in comparison with CLC) and materials (epoxy resin sealer: AH Plus/AH26 vs any other type of sealer) applied for treatment of apical periodontitis [56]. Nevertheless, in this study [56] high risk of bias was detected, thus the obtained findings should be interpreted with caution.

Some limitations of the present study should be acknowledged. Firstly, retrospective studies may be more biased than prospective studies because they allow for the selection of the cases included in the analysis. Consequently, randomized controlled trials or cohort studies on the clinical outcome of CSBSs should be performed [42]. In the present study, two sealers (only one CBCS) were used. Therefore, other sealers should be tested to provide a broader perspective on the investigated issue. Secondly, involving the operator in the evaluation, as it was in the present study, may potentially result in a subjective and more favourable evaluation [24]. The analysed index concerns periapical X-rays, which may result in an incomplete assessment of healing. Therefore, further studies using 3D imaging techniques (CBCT) should be conducted. Additionally, further research should examine the type of final reconstruction and its influence on treatment success. Moreover, in the above study, the treatment was performed by an endodontic specialist. Therefore, further studies are needed to evaluate the clinical effectiveness of treatment provided by general practitioners. Finally, in the present retrospective study, the average follow-up of the selected cases amounted to 7.56 months. The observation period should be longer to evaluate the retention of success achieved. However, it becomes more difficult to monitor patients over time due to the decreasing retention of participants in clinical trials.

## Conclusions

According to the results of this study, the success of treatment was comparable for both sealers, suggesting AH Plus Bioceramic Sealer as an alternative to gold standard epoxy-resin sealers, with risk factors including preoperative diagnosis and treatment type.

## Data Availability

No datasets were generated or analysed during the current study.
